# Potential Chemicals from Plastic Wastes

**DOI:** 10.3390/molecules26113175

**Published:** 2021-05-26

**Authors:** Ravindra Prajapati, Kirtika Kohli, Samir K. Maity, Brajendra K. Sharma

**Affiliations:** 1 Distillate and Heavy Oil Processing Division, CSIR-Indian Institute of Petroleum, Dehradun 248005, India; ravimprajapati@gmail.com (R.P.); kirtika.kohli@iip.res.in (K.K.); skmaity@iip.res.in (S.K.M.); 2 Prairie Research Institute-Illinois Sustainable Technology Center, University of Illinois Urbana—Champaign, Champaign, IL 61820, USA

**Keywords:** plastic wastes, chemicals, chemical recycling, carbon nanomaterials, carbonization, biodegradable plastics

## Abstract

Plastic is referred to as a “material of every application”. From the packaging and automotive industries to the medical apparatus and computer electronics sectors, plastic materials are fulfilling demands efficiently. These plastics usually end up in landfills and incinerators, creating plastic waste pollution. According to the Environmental Protection Agency (EPA), in 2015, 9.1% of the plastic materials generated in the U.S. municipal solid waste stream was recycled, 15.5% was combusted for energy, and 75.4% was sent to landfills. If we can produce high-value chemicals from plastic wastes, a range of various product portfolios can be created. This will help to transform chemical industries, especially the petrochemical and plastic sectors. In turn, we can manage plastic waste pollution, reduce the consumption of virgin petroleum, and protect human health and the environment. This review provides a description of chemicals that can be produced from different plastic wastes and the research challenges involved in plastic waste to chemical production. This review also provides a brief overview of the state-of-the-art processes to help future system designers in the plastic waste to chemicals area.

## 1. Introduction

Plastic waste pollution is a major threat to ocean, wildlife, and human health. The global plastic market size was valued at USD 568.9 billion in 2019 and is expected to grow at a compounded annual growth rate (CAGR) of 3.2% from 2020 to 2027 [1]. However, the recent outbreak of Coronavirus (COVID-19) is taking the plastic waste pollution problem to a whole new level. Projections have shown that the global plastic packaging market is expected to grow from USD 909.2 billion in 2019 to USD 1012.6 billion by 2021 at a CAGR of 5.5%, mainly due to the pandemic response [2]. Most of this plastic waste ends up either in landfills or incinerators and is lost forever as a resource, despite its potential for reuse and recycling. According to the Environmental Protection Agency (EPA), in 2015, 9.1% of the plastic materials generated in the U.S. municipal solid waste stream was recycled, 15.5% was combusted for energy, and 75.4% was sent to landfills [3]. Plastic waste dumping creates serious difficulties in maintaining a clean and green environment. Yet, plastic waste reuse and recycling are projected to generate a profit-pool growth of USD 60 billion for the plastic and petrochemical sectors [4]. To generate profit, a petrochemical industry should establish a waste-collection system to adapt the plastic waste recycling strategies. In addition, plastic and petrochemical industries need to implement a different business model in which they can consider plastic waste supplies from various sources rather than obtaining raw material from one source. These industries should maintain product-portfolio priorities and implement a circular economy as much as possible [4].

Researchers worldwide have taken up plastic waste as an opportunity resource and are investigating innovative technologies that promote the recycling of plastic wastes. This includes research and development to produce new raw chemical materials and develop chemical recycling strategies to create value out of waste. The most common approach is converting plastic waste into a secondary raw material such as monomers or pyrolysis oil. The recycled material can be used in the production of new plastics, and the pyrolyzed oil can be fed into a chemical production unit (such as steam cracker); this way, these plastic waste products can replace fossil-based feedstocks.

**Scheme 1:**
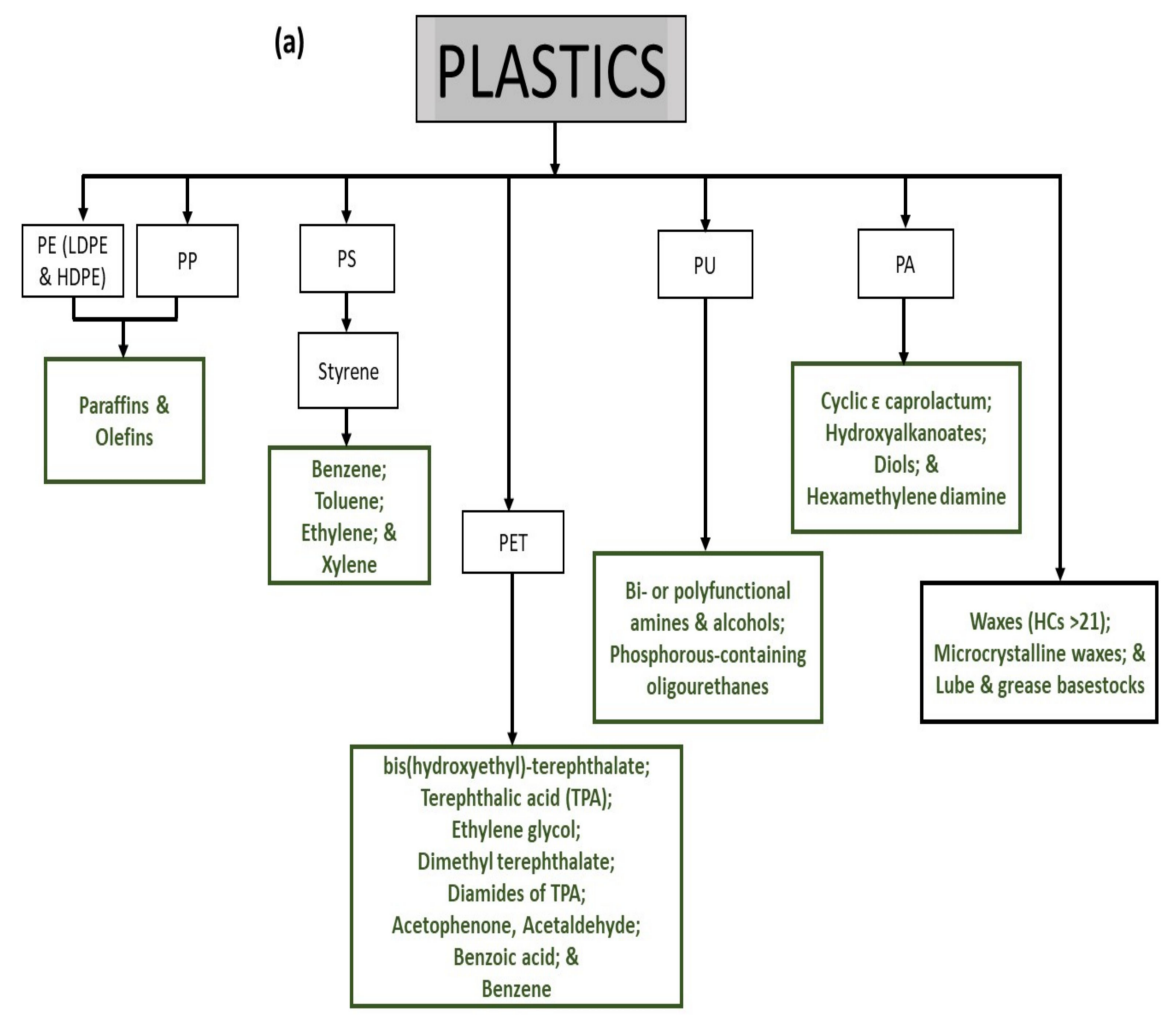

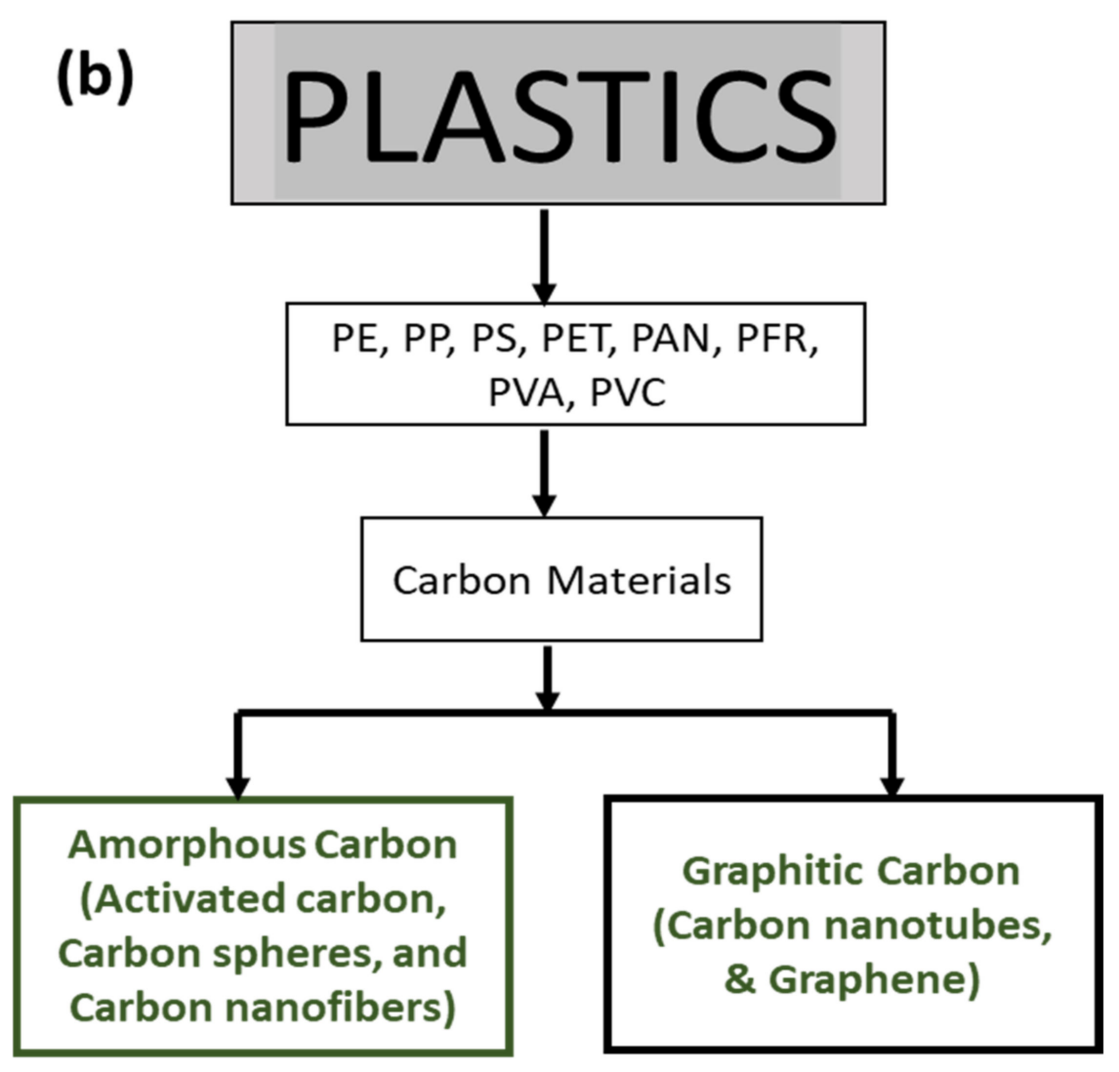
(**a**) Various chemicals and (**b**) carbon materials produced from plastics.

Lately, the research focus has been devoted to fuels such as hydrogen [5], gasoline [6], and ultra-low sulfur diesel [7] production from plastic wastes and recycled plastics. Moreover, cleaner fuels are the ultimate goal in such processes in which a high molar ratio of hydrogen to carbon is found [8]. A significant number of reviews have systematically and thoroughly discussed fuel production from plastic wastes [9,10,11,12]. Although the making of high-value chemicals from the liquid feedstock (generated from plastic wastes) is considered a breakthrough for hard-to-recycle plastics, this aspect has been reviewed less often. In the present scenario, especially in the COVID-19 pandemic situation, the demand for fossil fuels has become historically low, and it may continue for a while. Therefore, petrochemical industries need to put more emphasis on the preparation of other value-added products such as chemicals or chemical building blocks from plastic wastes. This review mainly discusses the type of chemical raw materials and chemicals that can be recovered from plastic wastes (shown in Scheme 1). Plastic waste can also serve as a carbon source to produce valuable carbon-based products because carbon is the main constituent of plastics. Therefore, a separate section about carbon materials that can be made from plastic wastes is included. In addition, the advantages and disadvantages related to the known processes are discussed. Finally, the challenges and future perspectives associated with converting plastic waste to chemicals are summarized.

## 2. Production of Chemicals from Plastic Wastes 

Chemical recycling incorporates sustainability principles because it can produce either new chemical raw materials or original raw materials. In chemical recycling processes, the pyrolysis method is considered a stand-alone facility for the upgradation of plastic wastes. This process is highly useful, particularly with polyolefins (POs), which contain 2/3 of the plastic wastes to produce gaseous or liquid fuels or raw chemicals, mainly light olefins and benzene, toluene, and xylene (BTX). Polyolefin pyrolysis has gained significant interest because pyrolysis can be performed in small units near the collection sites. Therefore, one can avoid the costs related to the transportation of plastic wastes. The products obtained by thermal pyrolysis at low and high temperatures from plastics are given in Table 1. A wide range of hydrocarbons (HCs), such as paraffins, olefins, and aromatics, can be produced from the pyrolysis of plastic wastes. The yields of these HCs depend on the chemical composition, structure, and decomposition of plastics. For instance, the pyrolytic product from polystyrene (PS) waste can be refined to produce styrene, while paraffins and olefins can be obtained from polyethylene (PE) and polypropylene (PP) wastes. Further, thermal pyrolysis of polymethyl methacrylate produces a monomer, i.e., methyl methacrylate, and a 98% yield was reported at 450 °C [13].

Pyrolysis–catalysis has proved to be a promising technology for the plastic waste conversion into high-value products. The catalysts used for the plastic waste conversion play an important role during the processing. In general, plastics do not degrade easily, due to the presence of very strong carbon−carbon bonds. Through catalytic means, we can regain the high energy that holds these bonds in plastics; this will help to convert the plastic wastes into value-added commercial products. POs are challenging to deconstruct catalytically. The catalysts consisting of nanoparticles could help to develop more robust and effective recycling methods. The catalytic hydrogenolysis of POs has been investigated using various catalytic systems. Highly electrophilic Zr-H species prepared by surface organometallic chemistry convert the high-molecular-weight polymers (Mw = 125,000 Da), with the C_20_−C_50_ carbon chain, into fuels and smaller HCs [14]. Pt, Ir, Ru, and Rh nanoparticles have been studied for the catalytic hydrogenolysis of C_2_−C_10_ alkanes [15,16,17]. The catalytic activity depends on various factors such as operating conditions, the degree of substitution at each carbon atom of n-alkanes, and the characteristics (size and metal type) of supported metal particles. The product distribution also depends on the feedstock properties. For instance, with the Ni/H-beta catalyst, a high yield of gasoline and light diesel was obtained from PP rather than low-density polyethylene (LDPE) or high-density polyethylene (HDPE) [18]. However, in another report, more aromatics were produced from HDPE compared to LDPE [19]. In addition, carbon nanomaterials that can be recycled from plastic waste have also attracted attention in recent times. More details about carbon nanomaterials are provided in Section 3.

Another promising technology is hydrothermal liquefaction (HTL). It is highly flexible in treating both pure and mixed waste streams. The HTL technique is based on fast-heating-rate reactors with moderate residence times (15–20 min), temperatures (300–360 °C), pressures near to water saturation, and the use of catalysts (based on the feedstocks) [20,21]. Passos et al. [21] demonstrated a total of 12 different commercial polymers such as acrylonitrile-butadiene-styrene (ABS), HDPE, LDPE, polyamide 6 (PA6), polyamide 6/6 (PA66), polyethylene terephthalate (PET), polycarbonate (PC), PP, PS, and polyurethane foam (PUR) using the subcritical HTL process. The HTL reactions were performed in a 20 mL autoclave reactor at 350 °C for 20 min. The main findings were as follows: (i) bis-phenol-A (BPA) and its derivative compounds were identified in the oil products from epoxy and PC polymers; (ii) solid terephthalic acid (TA) as the major product was obtained in noncatalytic HTL of PET; (iii) from PA6 and PA66, AP monomers were produced, and these monomers can be repolymerized, if pure feeds are used; (iv) the oil produced from PUR polymer is a complex that contains oligomers and low-boiling-point compounds; (v) the solid residues from PVC are highly dechlorinated, and this fraction can be used as a carbon source. The results suggested that each type of synthetic polymer undergoes a different type of depolymerization based on its composition under HTL processing.

Gasification is another process that can produce syngas, which can be used as a precursor to produce acetic acid, methanol, aldehyde, carbohydrates, ammonia, etc. This process is the most advantageous because it can treat even nonsegregated wastes. However, the process produces poisonous hydrogen cyanide and nitrogen oxide as gases, and the emissions can be reduced by using effective catalysts to some extent.

### 2.1. Polyethylene (PE) and Polypropylene (PP)

Table 2 summarizes the few recent reported studies for chemical production from plastic wastes, which are discussed in detail. In general, a conversion process of plastic yields gas, liquid, and solid residues. From PE and PP, liquid products in the range of 83 to 96% can be obtained by pyrolysis [22,23]. As said earlier, the composition of the final products depends on the type of feedstock, conditions used, catalytic or noncatalytic, reactor system, etc. [24]. In PE pyrolysis, the yields of aromatics increased from 3 to 6% and the yields of naphthalenes decreased from 22 to 17% [24], whereas in PP pyrolysis, the paraffins yield decreased from 33 to 27% with increasing temperature from 350 to 520 °C, and that of aromatics increased from 0.8 to 11% with increasing temperature from 350 to 600 °C. A two-step process involving pyrolysis and downstream catalytic cracking was applied for the light olefin production from HDPE, and the pyrolysis was performed in a conical spouted-bed reactor (CSBR) at a reaction temperature of 500 °C. The volatile stream obtained from the HDPE pyrolysis in a CSBR mainly contained waxes (>C_21_), and this volatile stream was passed through a fixed-bed (downflow) catalytic reactor in the presence of HZSM-5 zeolite. It was found that 67% of the waxes were converted into light olefins. This is because of the shape selectivity, low hydrogen transfer capacity, and moderate acid strength of the HZSM-5 zeolite [25]. Besides the acidity of the HZSM-5 zeolite, the short residence time in the reactor was found to increase the selectivity of the light olefins and decrease the coke formation. The high-value aromatic chemical raw materials such as benzene, toluene, and other aromatic HCs can also be obtained by refining the pyrolytic product. For instance, the pyrolysis of PE and PP produces a liquid product that mainly contains BTX compounds [26]. The BTX yield can be increased by increasing the reaction temperature and using suitable catalysts. In general, aromatic compounds are formed due to secondary reactions and shape selectivities of the catalysts. Zhang et al. [27] developed a low-temperature catalytic method to convert PE directly into liquid alkylaromatics using a Pt/γ-Al_2_O_3_ catalyst. The produced alkylaromatics have applications such as lubricants, surfactants, insulating oils, and refrigeration fluids.

### 2.2. Polystyrene (PS)

PS, a thermoplastic, is used mainly in electrical appliances, medicines and packaging materials, thermal insulation, and in the automotive industry. In 2012, the U.S. generated ~2 million tons of PS waste, of which only 0.9% was recovered [42]. PS chemical recycling is mainly performed by the pyrolysis process [28,29,30,31,32,42,43,44,45]. A styrene monomer with high selectivity can be obtained via PS pyrolysis via thermal and catalytic routes. A 63% yield of styrene at 477 °C was observed [24]. This is because of the polycyclic nature of PS and the thermodynamic limitations in converting cyclic structures to aliphatic compounds. The product oil containing 83% (*w*/*w*) styrene was generated by PS pyrolysis at 520 °C using a fluidized-bed reactor [28]. With added organic additives such as naphthalene in the PS pyrolysis, the styrene yield can be enhanced [29]. Catalytic pyrolysis using ZSM-5 zeolite produces oil, which mainly contains single-ring aromatics such as ethylbenzene and toluene [43]. Zhang et al. [30] reported that the various basic catalysts helped to increase the monomer yield compared to thermal and/or acid-catalyzed pyrolysis. In addition to aromatic chemicals, the direct re-polymerization of the PS pyrolysis product to synthesize a polymer comparable to the original PS was also reported [31]. Productions of monomers such as benzene, toluene, ethylene, and xylenes (BTEX) were reported by using the two-stage pyrolysis process, which includes an auger and a fluidized-bed reactor [32]. A high value, i.e., 26%, of BTEX was obtained [32]. The microwave-assisted pyrolysis of PS with coal was investigated, and aromatic liquid products in the narrow range with acetylene and hydrogen sulfide were produced [44].

### 2.3. Polyethylene Terephthalate (PET)

**Figure 1:**
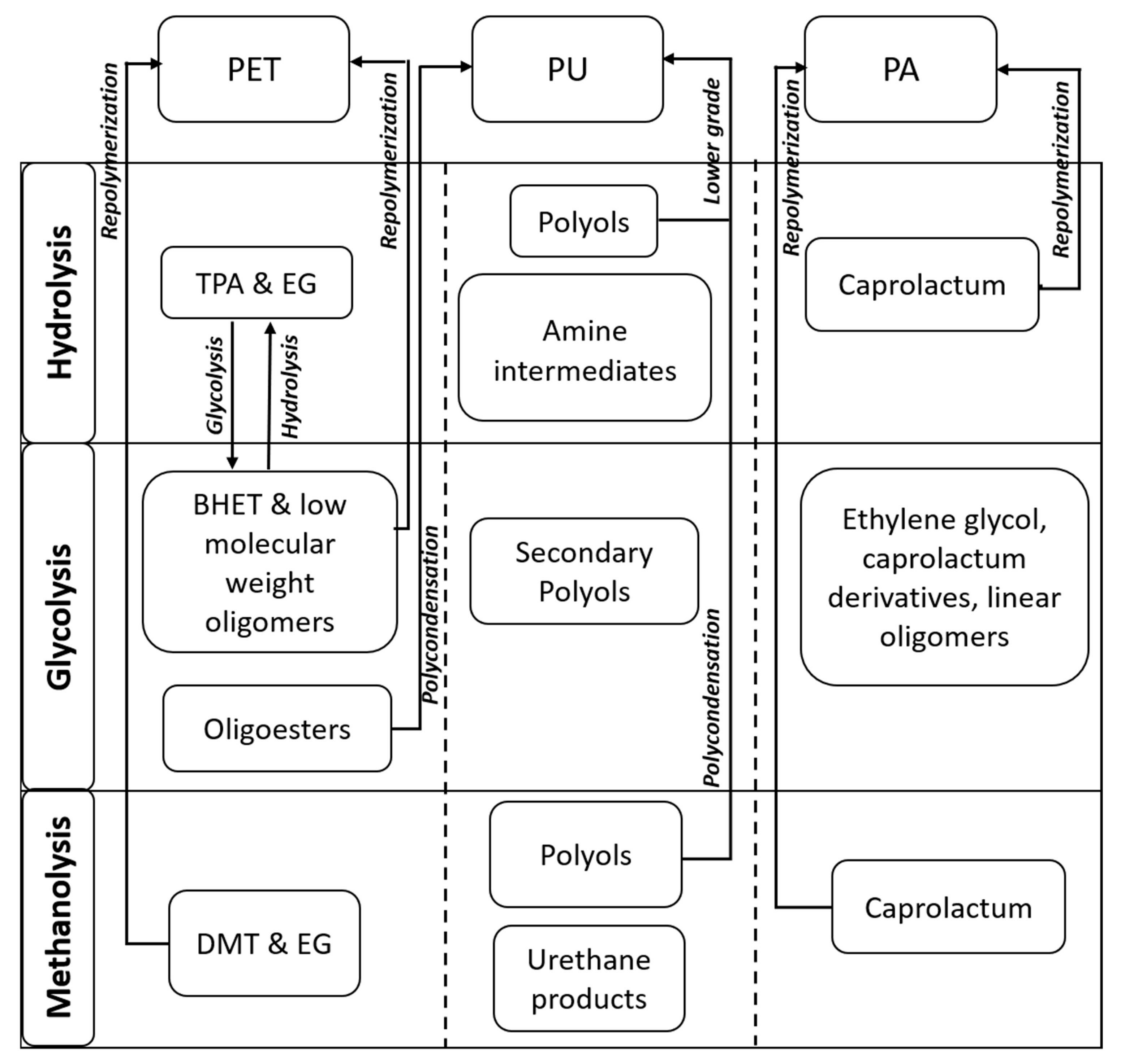
Products obtained through different pathways of polyethylene terephthalate (PET), polyurethane (PU), and polyamide (PA) (TPA = terephthalic acid; EG = ethylene glycol; BHET = bis(hydroxyethyl)-terephthalate; DMT = dimethyl terephthalate).

Polyethylene terephthalate (PET) is one of the top four thermoplastics used throughout the world primarily in food packaging, the textile industry, and the production of bottles [45]. The production of PET worldwide in 2014 was approximately 41.6 million metric tons (MMT) and is forecasted to be approximately 73.4 MMT by 2020 [46]. In the U.S., the recycling rate for PET packaging was 31.2% in 2013, according to the Association of Postconsumer Plastic Recyclers (APR) and the National Association for PET Container Resources (NAPCOR). The recycling rate of PET waste is very low; therefore, there is a need to develop economical and low-carbon-footprint depolymerization processes to utilize this plastic waste in value-added applications. Chemical recycling methods for the PET wastes consist of glycolysis [33], hydrolysis [34], methanolysis [35], and aminolysis [36]. The most commonly used method is glycolysis because it is a simple, flexible, and low-cost process. In the glycolysis process, PET is treated by a glycol such as ethylene glycol (EG), propylene glycol, diethylene glycol, and triethylene glycol (TEA), with transesterification catalysts to yield bis(hydroxyethyl)-terephthalate (BHET) (shown in Figure 1) [33]. The produced BHET can be used with virgin PET or can be utilized for different PET production processes. The BHET yield and glycolysis reaction rates depend on the different reaction parameters such as temperature, type and amount of catalysts, and the PET/glycol ratio. On the contrary, via the hydrolysis method, terephthalic acid (TPA) and EG are mainly produced at high pressure (1.4–2 MPa), high temperature (200–250 °C), and longer reaction times [31]. Hydrolysis can be acidic (sulfuric or nitric acid), alkaline (sodium hydroxide), or neutral (metal catalysts). The cost associated with the process is high, and therefore, this process is not commercially used. The next method is methanolysis (treatment with alcohol), in which dimethyl terephthalate (DMT) and EG are mainly produced [35]. Finally, the aminolysis method involves the reaction of PET with amines such as allylamine, morphine, hydrazine, and polyamines to produce diamides of terephthalic acid (TPA) [36]. In recent times, the treatment of PET wastes with different amino alcohols, ethanolamine, has been of significant interest. This process leads to the production of phthalimide diols (low-cost polyols). The solid powder polyols (terephthalate diol) produced after the aminolysis step can be used as a building block to produce different kinds of polyurethane (PU) products with a higher economic value. PUs are the most important elastomers with extensive industrial applications. The global polyurethane market is forecasted to increase to USD 74.24 billion in 2021 from USD 58.28 billion in 2018 [47].

PET pyrolysis products consist of various aromatic and oxygenated compounds such as vinyl benzoate, benzoic acid (BA), and acetaldehyde. PET pyrolysis using ZSM-5 zeolite and NiCl_2_ catalysts was found to be effective for producing more liquid products [37]. The pyrolysis of waste PET takes place by cleavage of the ester linkage, leading to the formation of vinyl ester and carboxyl compounds, mainly BA. The produced vinyl ester can be decomposed into compounds such as acetophenone, acetaldehyde, and lighter HCs (C_1−_C_3_) [48]. BA, which is a high-value chemical around USD 4000/ton [49], is mainly used in the food and beverage industries. BA is also used as a feedstock for manufacturing phenols, benzoates, and other antifungal preservatives. Besides, BA is used as a feedstock for fungal ointments (medical use), plasticizers, and as a calibrating material for bomb calorimeters [50]. Thus, the recovery and production of BA from waste PET can produce a potential chemical. Dimitrov et al. [38] demonstrated that in the presence of a different medium or contaminants, different pyrolyzed products can be obtained. For instance, when the pyrolysis of PET is performed with acidic contaminants, CO_2_/acetaldehyde, BA, and vinyloxycarbonyl benzoic acids are formed. While in the presence of a base, tetramethylammonium hydroxide (TMAH), dimethyl terephthalate, short-chain alcohol, and trimethylamine (TMA) can be produced. TMA and short-chain alcohol are formed from the dissociation of TMAH. In another study, TPA was produced from the pyrolysis of PET, which can later be converted to benzene in the presence of CaO under controlled conditions.

The use of mechanically recycled PET as an additive in asphaltic mixtures has been explored. Modified asphalt prepared using PET wastes has shown advantages with respect to rutting and fatigue parameters and creep deformation as well [51,52]. Merkel et al. reported the use of chemically deconstructed mixed PET waste as an additive for asphalt [53]. The proposed approach utilizes the aminolysis process in which PET waste was treated with various amine nucleophiles to generate terephthalic amides with distinct structures such as polar, nonpolar, and lipophilic. For the activity demonstration, the generated terephthalic amides were added to the road-grade asphalt binder at 5 wt.% and the performance was investigated. Parameters such as rutting, fatigue characteristics, and thermochemical and creep performances were evaluated. The results revealed that the addition of these additives increases the performance by as much as 18%. Asphalt, mainly used in road construction and roofing, is the most expensive part of the road-paving material, although asphalt makes up only 5 wt.% of the pavement mixture. However, the cost of asphalt was approximately USD 610 per ton in 2012 [54]. Thus, recycling PET waste can produce high-performance asphalt paving mixtures.

### 2.4. Polyurethane (PU)

Polyurethane (PU) represents 8% of the total plastics, mainly used in coatings, adhesives, sealants, elastomers, mattresses, and automotive seats. Chemical compounds such as polyols and amine intermediates can be produced from PU (flexible foams) hydrolysis. The hydrolysis process is difficult to use at a larger scale because of the use of high temperature and high pressure. This process is also uneconomical because the time taken for hydrolysis reaction is quite long, conversion is relatively low, and product purification is challenging. The reaction of PU foams in the presence of water, glycols, and basic catalysts is widely used [39,55,56,57]. Multifunctional alcohols and amines can also be obtained by processing PU with diamines or amino alcohols. For this process, PU is dissolved in suitable solvents such as cyclic ether, a chlorinated HC solvent, or N-methyl pyrrolidone. The reaction temperature for this reaction ranges from 200 to 210 °C with catalysts [39]. Phosphorous containing oligourethanes can also be produced by treating PU with esters of phosphoric and phosphonic acids [58]. This technology has been less explored. These oligourethanes can be used to make new PUs with enhanced flame retardant, UV resistance, and adhesive properties.

### 2.5. Polyamides (PA)

Polyamides (PA) are utilized for various applications such as fibers in carpets and textiles, electrical and electronic industries, engineering plastic in the automotive and construction industries, and the coating and packaging sectors. Cyclic ε-caprolactam (CPL) as a monomer can be recovered from PA depolymerization (Figure 1). The depolymerization of PAs is mainly carried out by alcohols/glycols, ammonia, water, and in the presence of catalytic agents [59,60]. The major challenge associated with PA depolymerization is the harsh reaction conditions, which lead to the formation of undesired side products that create problems in purification. For instance, a 78% CPL yield was obtained from PA6 by hydrolysis in the presence of phosphotungstic acid at a reaction temperature of 300 °C and reaction time of 85 min. Products such as 6-amino-caproic acid and water-soluble oligomers were produced as side products [60]. PA glycolysis using EG with a diammonium hydrogen phosphate catalyst at 190 °C for 1.5 h led to incomplete degradation. A blend of glycosylates obtained was used as a replacement for industrial polyols in PU production [61]. The combination of diols and diesters was also produced from PA-based wastes in supercritical methanol at 330 °C [62,63]. Aminolysis can convert PA 66 and PA 6 plastics to hexamethylenediamine (HMDA). This occurs via the conversion of carboxylic groups through the amides to nitrile, and then these can be hydrogenated to provide a final amine group [40]. Cesarek et al. [41] demonstrated the use of microwave irradiation for the efficient depolymerization of PAs into a monomer without any side-product formation. The complete hydrolysis of PAs was demonstrated at a temperature of 200 °C in a relatively short time, and the high-quality monomers were recovered.

The production of waxes from plastic wastes has also been reported. These waxes have some special characteristics compared to waxes obtained directly from petroleum. The unique characteristics are excellent distribution, smooth flow behavior, high softening point, chemical- and water-resistant properties, and better chemical stability. The waxes produced have a large market and are used for applications such as an antioxidant additive for rubber, candles, shine products for wood floors and cars, paint cans, lubrication, and as an additive in the fabricating and processing of POs. The waxes can also be used for asphalt roads [64,65] and roofs and as additives for plastics, coatings, and adhesives. Wax as high as 90 wt.% can be produced from PE under suitable pyrolysis conditions [66]. HDPE pyrolysis produced waxes (HCs > C_21_) selectively using a CSBR reactor at a 500 °C reaction temperature [26]. The production of high-value chemicals such as different grades of microcrystalline wax, paraffin wax, and lube and grease base stocks were reported by the conversion process, including low polymer wax or polymer mud [67]. These low polymer waxes were obtained as a by-product during HDPE production. The conversion involves thermal treatment in the presence of organic peroxides, such as butyl peroxide and benzoyl peroxide, and metal oxides such as magnesium oxide and calcium oxide. The product composition was found to be dependent on the process parameters used, such as the type of peroxides, metal oxides, reaction temperature, and reaction time. Low polymer waxes can also be converted to gasoline, diesel, and aromatics along with liquified petroleum gas (LPG) via a conversion process that includes pyrolysis followed by a vapor-phase catalytic conversion in the presence of zeolites. The products obtained from the reaction contain HCs in the range of C_5−_C_16_. Celik et al. [68] developed a stable nanoparticle-support catalyst for the upcycling of single-use polyethylene into high-quality liquid products. The developed catalyst consisted of strontium titanate (SrTiO_3_), an archetypical cubic perovskite, as a support for the deposition of PtNPs to form a Pt/SrTiO_3_ catalyst. The used SrTiO_3_ was single crystal nanocuboids having a sub-100 nm average size, with {100} facets and rounded stepped edges. The hydrogenolysis was performed at 300 °C and 170 psi of H_2_ under solvent-free conditions. The results suggest that PE adsorption is more favorable on Pt sites compared to the SrTiO_3_ support. Pt edge sites were found to be highly reactive for PE hydrogenolysis compared to Pt facets.

The production of high-quality lubricating oils from plastic waste has also been investigated by a few researchers [27,69]. Lubricating oil without added additives is called base oil and has a viscosity index (VI) in the range of 95–105; these are called conventional base oils. Base oils with VI values > 115 are known as unconventional base oils (UCBO). Miller [69] developed a new process for the conversion of plastic waste and Fischer–Tropsch (FT) wax to lube range molecules, and these can be hydroisomerized to low-pour-point base oils with UCBO quality. Different types of feedstocks were used such as PE, 96% PE + 4% PET, FT wax, and a 50/50 mixture of PE and FT wax. In this work, the pyrolysis process converts high-molecular-weight compounds into lower-molecular-weight compounds in the lube oil range HCs. After pyrolysis, the hydroisomerization process was used to produce low-pour-point oils of UCBO quality. The authors reported that hydrotreatment of feed prior to the hydroisomerization step did not significantly affect the lubricating oil yield and quality.

Overall, the recovery of chemicals from plastic wastes is challenging because of the difficulty in separating catalysts (mainly homogeneous catalysts) from the products and purification from other products. Another difficulty is the slow reaction rates with low selectivity that generate significant challenges in scaling up to a commercially applicable process.

## 3. Production of Carbon Materials

Plastic wastes can be converted to carbon materials such as amorphous and graphitic carbon. The amorphous carbons include mainly activated carbon, carbon spheres, and carbon fibers, while carbon nanotube (CNT) and graphene are graphitic carbon materials [70,71]. A two-step process, i.e., pyrolysis followed by carbonization, is generally used to make these carbon materials, and these processes are collectively termed carbonization. These processes produce various HC gases and a residual product with a high amount of carbon (carbon materials). Carbonization processes are usually performed under different conditions and are categorized as anoxic pyrolysis, catalytic, and pressure carbonization, as described by Chen et al. [72]. Table 3 summarizes the different carbonization processes that can be used to make carbon materials from plastics.

In the anoxic pyrolysis process, plastics are treated at a high temperature in an inert atmosphere under atmospheric pressure. The carbon atoms present in plastics are converted into carbon materials during the heat treatment process by an aromatization mechanism. Gases such as NH_3,_ CO, CO_2_, HCl, CH_4_, and H_2_ are also formed. Phenol-formaldehyde resins (PFR) are mainly used to produce amorphous carbon in an inert environment using a high-temperature pyrolysis process. This process produces a 62 % carbon yield at a 1000 °C reaction temperature [73]. The carbonization of PET alone or mixed with coal/pitch produces high-quality activated carbon [74]. LDPE plastics can be transformed into carbon composites. To make carbon materials from LDPE, a thermo-oxidative process in the presence of air at a temperature of 270–330 °C is generally used [75]. The chemical stabilization processes are also applied for POs and PS before making carbon materials via carbonization. For instance, the chemical stabilization of PE and PS is performed by the sulfonation process with sulfuric acid or chlorosulfonic acid [76]. Then, the sulfonated PEs are easily converted to carbon materials via simple heat treatment in an inert environment.

Catalytic carbonization of the plastics is performed in the presence of catalysts such as transition metal materials and solid acids to obtain carbon nanomaterials such as CNTs, graphene, and carbon spheres. Catalysts containing iron (Fe) and/or nickel (Ni) such as iron hydroxide, Ni metal, ferrocene, and stainless steel are generally used to convert plastics to carbon materials. Recent studies in the literature revealed that the CNTs can be prepared from the pyrolysis of hard plastics, PFR, the most challenging type of plastic waste to be recycled. A two-stage pyrolysis-catalytic method was applied to produce CNTs from PE resin using Fe- and Ni-based catalysts [77]. The presence of active metal catalyst particles is required for the CNT formation; CNTs produced by Fe-based catalysts show better smoothness and possess a clear internal structure. Transmission electron microscopic (TEM) images show that CNTs have a diameter of 15–20 nm with a length of several microns. The results showed that with Fe catalysts, a 34% CNT yield (with 97% purity) can be obtained.

Table 4 summarizes the recent studies to generate carbon-based materials from polymeric wastes. Bimetallic catalysts such as Ni-Cu, Ni-Fe, Fe-Mo, and Mn-Fe showed higher activities for CNT production [78,79,80]. The catalyst support materials were also found to play promising roles in CNT production from plastic wastes. Yao et al. [78] utilized the different silica-alumina support materials such as ZSM5, MCM41, NKF5, and H-beta zeolite as the support materials to prepare Ni-Fe based catalysts. The catalyst activity for the decomposition of plastic into carbon followed the order Ni-Fe/MCM41 > Ni-Fe/ZSM5 > Ni-Fe/Beta > Ni-Fe/NKF5. Ni-Fe/MCM41 showed the highest activity due to the high surface area and abundant mesoporous structure, which increases the interactions between reaction intermediates and catalyst active sites. In another study, the effects of catalysis temperatures such as 600, 700, and 800 °C on carbon materials were evaluated [79]. The results suggested that the low catalysis temperature i.e., 600 °C, produced mainly amorphous and disordered carbons, while catalysis temperatures higher than 700 °C were found to be better for making the CNTs. However, a further increase in temperature mainly increases the yield compared to thermal stability and graphitic degree. The effect of Mn added to Fe-based catalysts was also investigated to produce CNTs [80]. The results revealed that an increase in Mn content from 0 to 10 wt.% promotes the CNTs yield. The authors reported that the dispersion of iron particles increased via the addition of Mn, inhibiting the sintering of iron particles.

Typically, pressure carbonization is carried out under high-pressure conditions and follows two routes: (1) direct carbonization, which is performed under the pressurized atmosphere of the decomposition gases; and (2) hydrothermal carbonization (HTC), which is performed in the presence of water vapor (>100 °C and 0.1 MPa). The pressure carbonization method could produce carbon materials (with micro- or nanostructures) with high yields compared to other carbonization techniques. The carbon yields and morphology were found to be strongly dependent on the polymer precursor used. For instance, the carbon spheres with a carbon yield of 45 wt.% can be obtained by adding 5–20 wt.% of polyvinyl chloride (PVC) to PE at a temperature of 650 °C and 30 MPa pressure. The carbon yield increased with an increase in PVC above 20 wt.% [81]. It is reported that PVC is transformed into carbon using the HTC process at a milder temperature. PP, which is more stable, can be converted into carbon composites via HTC at 220–250 °C in the presence of microwave radiation with 33–69% yields. The carbon yield is mainly dependent on the temperature and reaction time used for HTC [82].

Jie et al. [83] investigated the depolymerization of plastic waste (mixture of commonly used PP, PE, PP, and PS) using the microwave-assisted catalytic process into hydrogen and multi-walled CNTs. The results suggest that by using FeAlOx catalyst, a high production of 1560 mgC/g plastic/g catalyst with >92 wt.% multi-walled CNTs were produced.

The synthesis of carbon dots (C-dots) is also reported in the literature [84,85,86]. The C-dots generated from plastic wastes possess superior biocompatibility, adjustable luminescence, and optical properties. Chaudhary et al. [84] utilized plastic wastes consisting of used cups, bottles, and polyethylene bags and the hydrothermal carbonization process to make fluorescent C-dots. The produced C-dots consist of different types of functional groups such as −COOH and −OH on the exterior surface, and they possess better water solubility. In addition, the prepared C-dots act as nanosensors for the fluorescence quenching recognition of the Cu^2+^ metal ion pollutant. A carbo-catalyst of C-dots was prepared via the air oxidation and sulfuric acid sulfonation of PET [85]. The prepared C-dots comprise SO_3_H, COOH, and OH groups. TEM analysis showed that the prepared C-dots were well dispersed, with diameters ranging from 1 to 6 nm. Poly(lactide) (PLA) polymeric waste was utilized for generating photoluminescent C-dots, as reported by Lauria and Lizundia [86]. A one-pot hydrothermal reaction of water-soluble PLA oligomers was carried out to make spherical C-dots with diameters of 3 nm. The major findings from their studies are: (i) the catalyst and solvent-free industrial-scale generation of photoluminescent nanomaterials are possible from polymeric wastes; (ii) the molecular weight of the precursors plays an important role to synthesize C-dots; and (iii) it has been suggested that a few repeating units (10–20) are required to activate significant luminescence in the materials.

The production of carbon nanosheets (CNSs) via the carbonization of waste PP using the catalyst was reported, and the obtained CNS possessed a thickness of 4–4.5 nm and 62.8% yield. The catalyst used was prepared from ferrocene and sulfur [87]. Two-step pyrolysis processes at temperatures of 450 °C and 945 °C in an inert atmosphere were reported to obtain graphene nanosheets [88]. The synthesized nanosheets have been used for the fabrication of dye-sensitized solar cells and supercapacitors. The catalytic carbonization of PET waste in the presence of MgO/Co(acac)_3_ as a hybrid catalyst produced porous CNSs with a 36 wt.% yield [89]. The catalytic activity of the hybrid catalyst was much higher compared to MgO and Co(acac)_3_ itself. The results demonstrated the synergistic effect between Co(acac)_3_ and MgO.

In summary, though there is a substantial number of reports available in the literature, the large-scale application of the carbonization processes is still limited. The low carbonization that yields around 15–20 wt.% from the plastic waste limits their large-scale application. Therefore, it is still a challenge to efficiently carbonize polymeric wastes with high yield and high purity.

## 4. Biodegradable Plastics (BDP)

Biodegradable plastics (BDP) are a class of plastics that can be decomposed by microbes into organic compounds, water, and carbon dioxide (in deficiency of oxygen, methane instead of CO_2_). A subclass of BDP is compostable, and these can be biodegraded in a compost system. Very few of these plastics are domestically compostable; thus, the tag “compostable” generally implies that these are industrially compostable. The factors that affect the biodegradability of polymers are mechanical properties; chemical characteristics of the polymers such as structure, molecular weight, and molecular weight distribution; and surface characteristics like surface area, hydrophilic, and hydrophobic properties [90,91].

**Figure 2:**
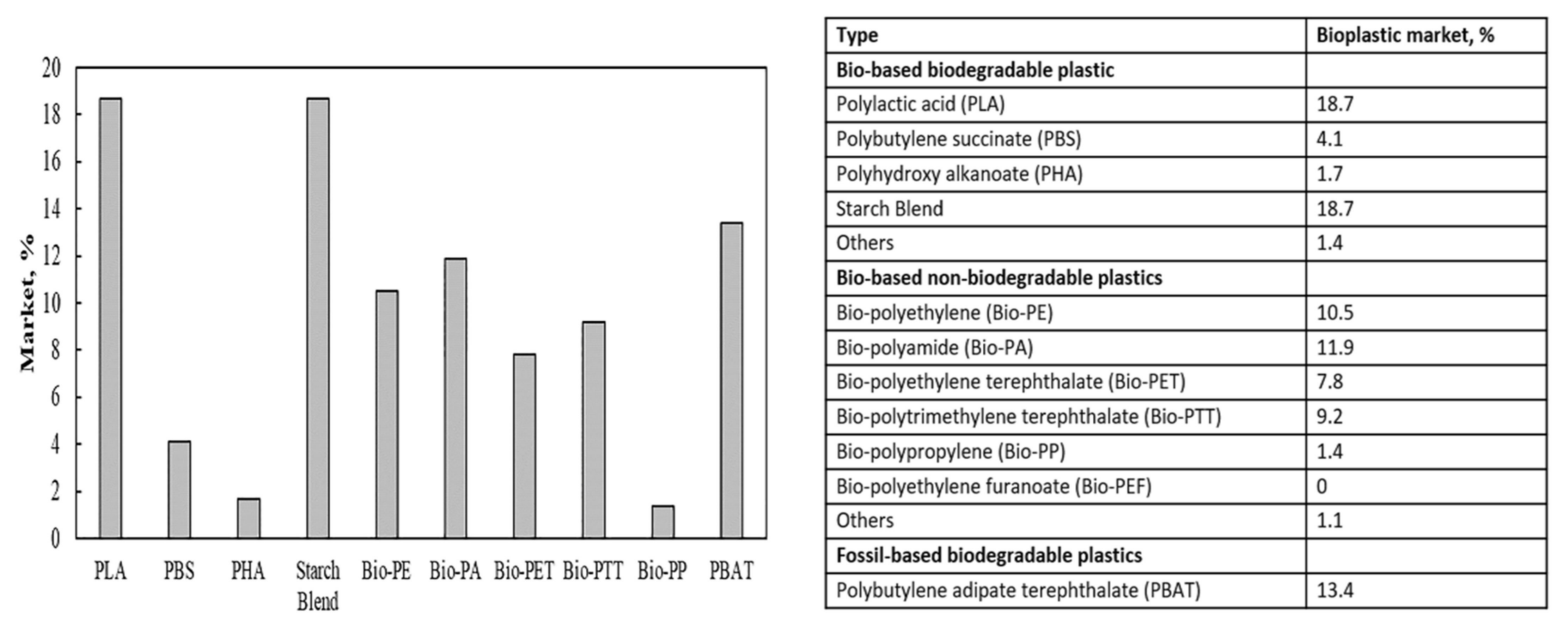
Bioplastic market in 2020 as per European Bioplastic data [94].

Bioplastics are plastics that are mainly made from biological sources such as biomass [92]. Biodegradable bioplastics can be either bio-based or fossil-based. However, bio-based bioplastics can be nonbiodegradable [93]. Presently, about more than 45 percent of the present bio-originated plastics are nonbiodegradable. Both biodegradable and nonbiodegradable bioplastics are presented in Figure 2.

Because of the advantages of bioplastic in the perspective of potential biodegradation and fossil fuel savings, the bioplastic demand is rising quickly. This will replace conventional plastics in the near future. According to the European Bioplastic Association, the worldwide production capacity of bioplastics is 2.11 million tons in 2020 [94]. It will increase to 2.87 million tons by 2025. Nonbiodegradable bio-based bioplastics are mainly polyethylene (bio-PE), polyethylene terephthalate (bio-PET), polyamide (bio-PA), polytrimethylene terephthalate (bio-PTT), polypropylene (bio-PP), and polyethylene furanoate (bio-PEF). Nonbiodegradable bioplastics are mainly prepared from bio-based resources such as biomass, sugarcane, and corn. The production process involves various steps such as pretreatment, hydrolysis, fermentation, and several organic reactions. Due to the growing awareness among the consumers, McDonald’s and several other enterprises have started utilizing bioplastic containers to provide their product to their customers. These bio-based plastics have a low carbon footprint and similar mechanistic properties to conventional plastics; still, their usage is very low. This might be due to the low cost of the petrochemical-based production process compared to renewable biomass resources. Rahman and Bhoi [95] recently reported a summary of nonbiodegradable bioplastics. The presented review also discussed the characterization of the bioplastic wastes (bio-PE, bio-PP, bio-PET, and bio-PA) based on the conventional plastic characterizations. Bio-PP and bio-PE can be used as feedstocks for the catalytic pyrolysis to produce gasoline and middle distillate HCs. On the contrary, bio-PET and bio-PA can become a potential feedstock for gasification processes, because of their higher oxygen content.

Table 5 presents a few examples of biodegradable polymers along with advantages and disadvantages associated with their use [96,97,98,99,100,101,102,103,104]. The biodegradable bioplastics include starch blend, polyhydroxyalkanoate (PHA), polylactic acid (PLA), and polybutylene succinate (PBS). Polybutylene adipate terephthalate (PBAT) is a fossil-based biodegradable polymer. Among the biodegradable polymers, starch blends are produced in large quantities followed by PLA. PLA contributes to 18% of the bioplastic market, and it has mechanical properties similar to PS. As a result, PLA could replace PS applications and could be a sustainable material [94]. PHA makes up 1.7% of the bioplastic market; however, their production is set to increase (quadruple) by 2023 [94]. PHA had excellent barrier properties similar to those PET, and better mechanical properties similar to those of LDPE. In addition, their monomers are highly variable, which allows for the tailoring of the finished polymer properties. As per reference [96], PBAT can be 50% bio-based and PBS can be 100% bio-based. Further, we discuss the two well-known biodegradable polymers (PLA and PHA) in the following text.

PLA is a commonly known compostable bioplastic. Its demand is continuously increasing due to its applications in the textile, 3D printing, biomedical, and food packaging industries [105]. The monomer lactic acid (LA), which is mainly produced from the fermentation of sugar, is used to produce PLA. From LA, PLA is produced via two routes, i.e., polycondensation or through the ring-opening polymerization [106]. PLA is a thermoplastic biopolymer, and its cross-linking of chains makes biodegradable plastic sheets that serve as the basis to produce various nonpolluting plastic products [107].

PLA is one of the most common bioplastics used today; however, its degradation process is very specific and needs appropriate facilities [108,109]. In general, the degradation of a polymer is classified as heterogeneous and homogeneous degradation, sometimes also called surface and intramolecular degradation. From a chemistry point of view, these can exist in three different ways: (i) scission of side chains, (ii) scission of the main chain, and (iii) scission of the intersectional chains. In PLA, decomposition occurs mainly by the scission of ester bonds, and longer polymeric chains are broken down into shorter monomer, dimers, or oligomers. In particular, the ester bonds in PLA are broken down into carboxylic acid and alcohol by chemical hydrolysis [108,109]. The degradation mainly occurs under aerobic conditions. The small fragments (in size) produced can pass through the cell walls of microbes and can be utilized as a substrate for their biochemical processes, and can be decomposed by microbial enzymes [108]. Zaaba and Jaafar [90] recently reviewed various degradation processes of PLA such as hydrolytic, photodegradative, microbial, and enzymatic. PLA degradation was found to be faster if PLA was immersed in 50% ethanol [110] because ethanol molecules diffused more rapidly within the polymer matrix compared to water molecules. Besides this, PLA hydrolytic degradation was reported using titanium dioxide (TiO_2_) nanoparticles [111], different temperatures [112], organic modifiers [113], epoxy-based chain extenders [114], and alkaline solutions [115]. The reduction in PLA molecular weight was found to be more effective with PLA/TiO_2_ nanocomposites compared to raw PLA. The results indicated that TiO_2_ nanocomposites act as nucleating agents, which changes the PLA crystallization behavior during the hydrolysis process [113].

PHAs are biobased and biodegradable polymers with multiple applications. These can be produced via bacterial fermentation, from substrates such as by-products from agriculture and the food industry [116]. PHAs possess thermoplastic properties that depend on the choice of feedstock, bacteria, and fermentation conditions used. Therefore, PHAs are ideal alternatives for conventional fossil-based plastics such as PE, PET, or PP. In addition, PHA can serve as an ideal candidate in the nanotechnology area and can find applications in various areas such as in the food and cosmetics industries, biomedicine, electrochemical sensors, and energy and environment. In addition, PHA can be blended with other polymeric materials and helps to improve thermal and mechanical properties relative to virgin plastics. PHA blends and nanocomposites are mainly used in biomedical applications. Most importantly, PHA blends and nanocomposites would be suitable alternatives for synthetic plastics so that these can be used as food packaging materials. A composite of PLA-PHB (75:25 concentrations) has remarkable optical miscibility and mechanical strength; these could be potential alternative for food packaging materials [117]. Arrieta et al. [118] synthesized PLA-PHB blends with catechin and showed that it can be used as a biobased packaging material for food industries. Amini et al. [119] described the synthesis of PHB/chitosan (nanofibrous) blends and effectively used it as a wound dressing material and cartilage tissue engineering. PHB with polyvinylidene fluoride nanofibrous membranes having antibacterial medications was utilized as a wound bandage. PHB was reported to be used as a drug carrier. For instance, Peng et al. [120] investigated PHB-PEG nanoparticles for the immobilization of insulin and its release. This could be used to treat diabetic patients. In another study, PHB cast films were reported to be used for cancer cell detection [121]. Researchers investigated the PHA nanofiber scaffold for the proliferation of neural stem cells, artificial blood vessels, and heart valves [122,123].

Despite several reports addressing the development in BDP polymers, the use of these polymers has faced several challenges, resulting in limited production and applications. The BDP plastics manufacturing cost is almost double compared to conventional plastics, which makes it price-competitive on the market. Due to the cost and mechanical properties of the known BDP polymers, these are blended with conventional plastics and chemical additives are added to meet product needs. These chemical additives might have negative impacts on health and the environment. Thus, evaluating the risks, true green, and eco-friendly nature of the BDP plastics is the most important challenge for the industries. Another challenge is the biodegradation and waste management facilities. The in-situ biodegradation of BDP plastics would require controlled and proper waste management facilities, which do not exist in many countries. To solve the plastic pollution problem in the true sense, we need the support of disposal infrastructure.

## 5. Research Challenges and Future Perspectives

A wide range of valuable chemicals such as paraffins, olefins, naphthalenes, benzene, toluene, xylene, carboxyl compounds, microcrystalline waxes, and lube and grease base stocks can be produced from plastic wastes. The state-of-the-art processes based on operating conditions play promising roles in the production of these chemicals. The limitations of the large-scale application are the harsh reaction conditions, aggressive reagent, low reaction rates, low chemical yields, and high purification steps involved. Innovative technological solutions including microwave treatment, plasma pyrolysis, and supercritical extractions using different solvents need to be explored. The most challenging part is the recovery of the chemicals because of the difficulty in the separation of catalysts from the products, as well as the purification from other products. A high-grade purified monomer is essential for the further polymerization reaction. Innovative solutions including nanoparticles, ionic liquids, or deep-eutectic solvents as catalysts should be considered. These catalysts promote the depolymerization reaction and a better yield of monomers.

Carbon materials such as amorphous and graphitic carbon can also be synthesized from plastic wastes. The recovery of carbon from the commonly used plastics is less popular and most of the carbon atoms mainly escape in the gaseous products. This leads to a large waste of energy and creates severe environmental pollution. Hence, the key to making carbon materials from plastic wastes is to increase the recovery of carbon to a significant level. Typically, various types and shapes of plastics are discarded such as fresh-keeping films, plastic stacks, rubbers, textiles, and electrical shells. These wastes need identification and sorting, which is also very challenging. The development of simple, cost-effective, and energy-efficient carbonization processes is required to treat plastic wastes without sorting and cleaning them. If the carbonization processes are successful on a larger scale, the carbon materials would be produced at least at a thousand-metric tons scale. If we are successful in making a significant amount of carbon materials, there will be an urgent need to explore new and practical applications. Traditional applications are in adsorbents, electrode materials, and catalysts. Novel uses of carbon materials can be in the production of solar steam, soil remediation, and biofilm precursors for treating contaminated wastewater.

## Figures and Tables

**Table 1:** Products from different plastics by thermal pyrolysis.

Polymer	Low-Temperature Products	High-Temperature Products
Polyethylene (PE)	Waxes, paraffin oil, alpha-olefins	Gases and light oils
Polypropylene (PP)	Vaseline, olefins	Gases and light oils
Polystyrene (PS)	Styrene and its oligomers	Styrene and its oligomers
Polymethyl methacrylate (PMMA)	Methylmethacrylate (monomer)	Low methyl methacrylate, more decomposition products
Polyethylene terephthalate (PET)	Benzoic acid (BA), Vinyl terephthalate	
Polyamide 6 (PA6)	ε-caprolactum (CPL)	

**Table 2:** Summary of reaction conditions, product yields, and key findings for chemicals produced from plastic wastes.

S. No.	Plastics	Method	Conditions	Product Yields	Key Findings	Source
1.	PE	Noncatalytic pyrolysis	T = 602 °C	Paraffins 45%; Olefins 32%; Naphthalenes 17%;Aromatics 6%;	A whole spectrum of HCs, including paraffins, olefins, naphthalenes, and aromatics.	[24]
	PP		T = 602 °C	Paraffins 27%; Olefins 36%; Aromatics 11%;		
	PS		T = 477 °C	Styrene 63%		
2.	HDPE	Thermal-catalytic two-step pyrolysis	T = 500 °C	Light olefin 59%	Higher efficiency of the two-step reaction system compared to the in situ catalytic pyrolysis (single-step) for production of 10 wt.% ethylene, 32 wt.% propylene, and 17 wt.% butenes.	[25]
3.	PP and PE	Fluidized bed reactor	T = 650–750 °C	BTX 32–53%	Higher feed rates and gaseous fluidizing medium have a positive effect on liquid oil production.	[26]
4.	PE	Mini-autoclave reactor (unstirred)	T = 280 °C, t = 24 h, Pt/Al_2_O_3_	Liquid product 80%	Tandem catalytic conversion produces a high yield of low-molecular-weight liquid/wax products.	[27]
5.	PS	Fluidized bed reactor	T = 520 °C	Styrene 83%	Complete conversion of PS to styrene oil was reported, with only traces of aliphatic compounds	[28]
6.	PS + organic compounds	Autoclave reactor	T = 400 °C, t = 1 h	Liquid 91%; Residue < 4%	Maximum styrene yield in the liquid was obtained with naphthalene as an organic compound with PS	[29]
7.	PS	Flow reactor	T = 350 °C, t = 3 h, Fe_2_O_3_	Liquid 83.6%; Residue 4.8% Styrene 74.3% (in liquid oil)	Barium oxide powder was found to be most effective catallyst for chemical recyling of PS waste	[30]
			T = 350 °C, t = 3 h, BaO	Liquid 93.4%; Residue 3.2% Styrene 76.4% (in liquid oil)		
			T = 350 °C, t = 3 h, HSM5	Liquid 78.2%; Residue 8.5% Styrene 64.4% (in liquid oil)		
8.	PS	Fixed-bed reactor	T = 510 °C thermal	Liquid 91.8%; Residue 5.7%	Other aromatic compounds can behave like a chain transfer agent and reduce the Tg of product polymer.	[31]
			T = 510 °C BaO (cat.)	Liquid 91.2%; Residue 8.1%		
			T = 510 °C FCC cat.	Liquid 90.7%; Residue 7.1%		
9.	PS	Two-stage auger and fluidized bed reactor	T = 780 °C	BTEX 26.3%	Product yields depend on the reaction temperatures and fluidizing mediums used.	[32]
10.	PET	Glycolysis	T = 190 °C; atm pressure	BHET 100% conversion, 84% yields	Lewis acidic ionic liquids [Bmim]ZnCl_3_ catalyst was found to be effective.	[33]
		Hydrolysis	T = 200–250 °C; P = 1.4–2 MPa	TPA, EG		[34]
		Methanolysis	T = 200 °C	DMT 64%; EG 63%	The product yields depend on the solubility of PET.	[35]
		Aminolysis	T = 70–110 °C	Diamides of TPA 66–89%	The bifunctional 1,5,7-triazabicyclo [4.4.0]dec-5-ene activates the carbonyl group and catalyzes the reaction.	[36]
		Pyrolysis	T = 450–600 °C ZSM-5 zeolite and NiCl_2_ used as catalyst	Aromatic hydroxyl groups increased by 22%	ZSM-5 facilitated the decomposition of carboxyl, aliphatic groups, and ether bonds in the primary products produced from the PET pyrolysis.	[37]
		Pyrolysis	T = 400–700 °C	Phenyl carboxylic acid 44–79%	Pd loaded on activated carbon used as a catalyst and produced more environmentally friendly products	[37]
11.	PET	Py-GCMS, EGA-MS, and TGA	T = 600 °C	4(vinyl oxy carbonyl) BA 27%; BA 10%	Wide range of liquid products obtained by different pyrolysis mechanisms.	[38]
12.	PU	Glycolysis	T = 200–210 °C; t = 2 h	Acetone-soluble products 80.8%; Residue 19%; Amines in total acetone soluble products 58.3 mgKOH/g	Polyol products produced from the process and used as initiators to produce oxy-alkylated polyols.	[39]
13.	PA 6 and PA66	Aminolysis	T = 100 °C; P = 3.5 MPa; t = 5.6 h; Raney^®^ Co 2724	ACN = 2; HMD = 32%; CPL = 46.2%; Other components = 13.6%	Raney^®^ Co provided a better catalytic activity along with long catalyst life	[40]
			T = 100 °C; P = 3.5 MPa; t = 5.6 h; Raney^®^ Ni 2400	ACN = 19.6; HMD = 15%; CPL = 46.5%; Other components = 14.7%		
14.	PA66	Microwave irradiation	T = 200 °C; t = 0.16 h; HCl:PA66 = 1:0.25	AA 90%; HMDA 86%; with 100% purity	The rate of PA hydrolysis depended on the PA type and HCl/amide molar ratio. With microwave treatment, high-purity and high-quality products were formed.	[41]

T = temperature; P = pressure; PE = polyethylene; HDPE = high-density polyethylene; PP = polypropylene; PS = polystyrene; PET = polyethylene terephthalate; PU = polyurethane; PA66 = polyamide 66; HCs = hydrocarbons; BTX = benzene, toluene, and xylene; BaO = barium oxide; FCC = fluid catalytic cracking; BTEX = benzene, toluene, ethylene, and xylene; BHET = bis(hydroxyethyl)-terephthalate; TPA = terephthalic acid; EG = ethylene glycol; DMT = dimethyl terephthalate; BA = benzoic acid; ACN = 6-aminocapronitrile; HMD = hexamethylene diamine; CPL = caprolactam; AA = adipic acid; HMDA = hexamethylene diamine; HCl = hydrochloric acid; py-GCMS = pyrolysis-gas chromatography mass spectroscopy; EGA-MS = evolved gas analyzer-mass chromatography; TGA = thermogravimetric analysis.

**Table 3:** Summary of the different carbonization processes and their carbon products.

Process	Operating Conditions	Plastics	Products
**Anoxic pyrolysis Carbonization**			
Without stabilization	T= 500–1000 °C in an inert atmosphere or in molten salt;	PET, PFR	Amorphous carbon products without metal impurities such as activated carbon, mesoporous carbon, and carbon fibers
Oxidation stabilization	Oxidation at T = 200–350 °C in the air; carbonization at T = 500–1000 °C in an inert atmosphere	PAN, LDPE, PVC,	
Chemical stabilization	Sulfonation or Friedel–Crafts reaction	PE, PS	
**Catalytic Carbonization**			
Catalytic carbonization	T = 400–900 °C in an inert atmosphere; with metal catalysts	PE, PP, PS, PVC, PTFE, PVA, PET, PFR	Graphitic carbons contain trace metals such as carbon nanotubes, carbon nanosheets, graphene, carbon foam
Catalytic pressure carbonization	T = 600–850 °C; with metal catalysts (in sealed reactor)	PP, PE, PS	Graphitic carbons
**Pressure Carbonization**			
Pressurized atmosphere	T = 600–850 °C (in sealed reactor)	PP, PE, PS, PVC PVC	Amorphous carbon such as carbon spheres, activated carbon, and carbon dots without metal impurities
Hydrothermal carbonization	T = 150–300 °C in the presence of water (in sealed reactor)		

PE = polyethylene; LDPE = low-density polyethylene; PP = polypropylene; PS = polystyrene; PVC = polyvinyl chloride; PVA = polyvinyl alcohol; PET = polyethylene terephthalate; PFR = phenol-formaldehyde resin; PAN = polyacrylic nitrile; and PTFE = polytetrafluoroethylene.

**Table 4:** Recent studies to produce carbon-based materials from polymeric wastes.

Plastics	Process	Catalyst	Reaction Conditions	Product Yields, wt.%	Source
Post-consumer plastic waste	Pyrolysis– catalysis	Ni-Fe/ZSM5	Catalysis T = 800 °C; Pyrolysis T = 500 °C for 15 min.	Carbon deposition = 50; Oil = 17; Gas yield = 37.80; H_2_ yield * = 35.80	[78]
		Ni-Fe/MCM41		Carbon deposition = 55.60; Oil = 16.30; Gas yield = 30.80; H_2_ yield * = 38.10	
		Ni-Fe/NKF5		Carbon deposition = 36.60; Oil = 27.40; Gas yield = 34; H_2_ yield * = 22.40	
		Ni-Fe/Beta		Carbon deposition = 47; Oil = 15.10; Gas yield = 32.10; H_2_ yield * = 32.80	
PP	Pyrolysis–catalysis	Ni-Fe	Catalysis T = 800 °C;	Gas = 48.5; Liquid = 20; Solid = 36 Carbon deposition = 360 mg/g feed	[79]
PP	-	10Mn-9Fe/Al_2_O_3_	T = 800 °C	Filamentous C = 32.89; Amorphous C = 8.69	[80]
PVC + PP	Pressure carbonization		T = 650 °C; P = 30 MPa	Carbon yield = 45	[81]
PP	Microwave assisted hydrothermal treatment	-	T = 250 °C; t =60 min	Amorphous carbon = 69	[82]
Plastic waste mixture	Microwave-assisted catalytic pyrolysis	FeAlOx	1000 W microwave power; t = 3-5 min	Carbon production = 1560 mgC/g plastic/g catalyst with 92% multiwalled CNTs	[83]
Waste Plastic (used cups, bottles, and PE bags)	Hydrothermal carbonization	-	T = 400 °C; t = 2 h	C-dot	[84]
PET	Air oxidation and acid treatment	-	Air Oxidation (T = 300 °C, time = 2 h); acid treatment (T = 120 °C, t = 6 h)	C-dots of diameters 1–6 nm	[85]
PLA	Hydrothermal carbonization		T = 180–240 °C; t = 4 h	C-dots of diameter 3 nm	[86]
PP waste		Ferrocene and Sulfur	T = 700 °C; t = 1.5 h	CNS = 62.8	[87]
Plastic waste (PP, PE, and PET)	Two-stage pyrolysis	Bentonite nanoclay	T1 = 450 °C; T2 = 945 °C	Graphene nanosheet	[88]
PET	Catalytic carbonization	MgO/Co (acac)_3_	PET:catalyst massratio = 1:2; T = 700 °C	CNS = 36	[89]

PE = polyethylene; PP = polypropylene; PLA = poly lactic acid; PET = polyethylene terephthalate; CNS = carbon nanosheets; C-dots = carbon dots; * = mmol H_2_g^−1^ plastic.

**Table 5:** Most used biodegradable plastics (BDP).

BDP	Feedstock	Properties	Limitations	Source
Polyhydroxyalkanoates (PHAs)	Microorganisms	UV stable, good humidity, and moisture resistance	Expensive and low thermal stability	[97]
Polybutylene succinate (PBS)	Succinic acid and 1,4-butanediol	Compostable, high flexibility, and outstanding thermal stability	Insufficient melt viscosity and stiffness	[98]
Lignin-based polymer composites such as polyethylene terephthalate (PET)	Lignin	Light weight, antimicrobial and environmentally friendly	High agglomeration	[99]
Polybutylene adipate terephthalate (PBAT)	1,4-butanediol, adipic acid, and dimethyl terephthalate (DMT) with 1,4-butanediol	Flexible and resilient	Low crystallization degree	[100]
Polylactic acid (PLA)	Starch	Compostable	Brittle and nonbiodegradable	[101]
Thermoplastic starch blend, for example, PLA/starch	Starch and plasticizers	Low cost	Brittle and low biodegradability	[102]
Cellulose bioplastic	Cellulose	Trouble-free processing	More costly	[103]
Polycaprolactones (PCL)	Petrochemical products	Very low degradation rate	Resistance to solvents	[104]

## Data Availability

The data presented in this study are available in this article.
